# Antitumor Effects of 5-Aminolevulinic Acid on Human Malignant Glioblastoma Cells

**DOI:** 10.3390/ijms22115596

**Published:** 2021-05-25

**Authors:** Mohammad Jalili-Nik, Farzaneh Abbasinezhad-moud, Sajad Sahab-Negah, Abolfazl Maghrouni, Mohammad Etezad Razavi, Maryam Khaleghi Ghadiri, Walter Stummer, Ali Gorji

**Affiliations:** 1 Epilepsy Research Center, Westfälische Wilhelms-Universität, 48149 Münster, Germany; mohammad.jalilinik@gmail.com (M.J.-N.); farzanehabbasinezhad@gmail.com (F.A.-m.); abol.maghrouni@gmail.com (A.M.); 2 Neuroscience Research Center, Mashhad University of Medical Sciences, Mashhad 9177948564, Iran; sahabnegahs@mums.ac.ir; 3 Student Research Committee, Mashhad University of Medical Sciences, Mashhad 9177948564, Iran; 4 Department of Medical Biochemistry, Faculty of Medicine, Mashhad University of Medical Sciences, Mashhad 9177948564, Iran; 5 Department of Neuroscience, Faculty of Medicine, Mashhad University of Medical Sciences, Mashhad 9177948564, Iran; 6 Shefa Neuroscience Research Center, Khatam Alanbia Hospital, Tehran 1996835911, Iran; 7 Ophthalmology, Eye Research Center, Mashhad University of Medical Sciences, Mashhad 9195965919, Iran; EtezadM@mums.ac.ir; 8 Department of Neurosurgery, Westfälische Wilhelms-Universität, 48149 Münster, Germany; Maryam.KhaleghiGhadiri@ukmuenster.de

**Keywords:** brain tumor, protoporphyrin, cell death, apoptosis, tumor cell line

## Abstract

5-Aminolevulinic acid (5-ALA) is a naturally occurring non-proteinogenic amino acid, which contributes to the diagnosis and therapeutic approaches of various cancers, including glioblastoma (GBM). In the present study, we aimed to investigate whether 5-ALA exerted cytotoxic effects on GBM cells. We assessed cell viability, apoptosis rate, mRNA expressions of various apoptosis-related genes, generation of reactive oxygen species (ROS), and migration ability of the human U-87 malignant GBM cell line (U87MG) treated with 5-ALA at different doses. The half-maximal inhibitory concentration of 5-ALA on U87MG cells was 500 μg/mL after 7 days; 5-ALA was not toxic for human optic cells and NIH-3T3 cells at this concentration. The application of 5-ALA led to a significant increase in apoptotic cells, enhancement of Bax and p53 expressions, reduction in Bcl-2 expression, and an increase in ROS generation. Furthermore, the application of 5-ALA increased the accumulation of U87MG cells in the SUB-G1 population, decreased the expression of cyclin D1, and reduced the migration ability of U87MG cells. Our data indicate the potential cytotoxic effects of 5-ALA on U87MG cells. Further studies are required to determine the spectrum of the antitumor activity of 5-ALA on GBM.

## 1. Introduction

Glioblastoma (GBM), the most aggressive and common cancerous primary brain tumor of astrocytic lineage, is an incurable and devastating disease with a median survival of 12 to 15 months [1,2]. The treatment plan usually includes initial surgical resection followed by concurrent radiation and chemotherapy [3]. Fluorescence-guided surgery with 5-aminolevulinic acid (5-ALA) relatively improves the poor outcomes of patients with GBM, presumably due to more complete resection of tumors and improvement in progression-free survival [4,5,6]. Fluorescence-guided surgery with 5-ALA is a valuable tool to enhance the extent of resection in GBM surgery, which significantly impacts prognosis in patients with GBM [7].

5-Aminolevulinic acid leads to selective intracellular biosynthesis and accumulation of protoporphyrin IX (PP-IX) in malignant glioma cells [8]. When irradiated with visible light, the tumor-specific accumulation of PP-IX results in cancer cell death and apoptosis via the production of reactive oxygen species (ROS) [9]. The co-application of 5-ALA and light at the appropriate wavelength enhances apoptosis in GBM cells by the inhibition of survival signal transduction pathways and amplification of the proteolytic activities [10,11]. Furthermore, 5-ALA effectively enhances the cytotoxic effect of multi-dose ionizing radiation on various neoplastic cells, including glioma cells [12,13]. In addition, 5-ALA-induced PP-IX accumulation may increase the cytotoxicity of ionizing radiation through the enhancement of ROS generation [14] and the modulation of cytokine and chemokine production [15]. Additionally, causal relationships between oncogene-mediated transformations such as human epidermal growth receptor 2 and Ras/mitogen-activated protein kinase and enhanced 5-ALA-induced PP-IX production have been reported [16,17]. Emerging evidence suggests that 5-ALA-induced PP-IX accumulation may affect the proliferation, migration, and invasiveness of GBM via the modulation of dynamin 2-mediated exocytosis [18,19]. Despite extensive investigations on the cytotoxic effect of 5-ALA in combination with phototherapy or ionizing radiation, it remains to be elucidated whether 5-ALA exerts a direct cytotoxic effect on GBM. In the present investigation, we examined the effect of 5-ALA at different doses on the viability, survival, and migration/invasiveness properties of the human U-87 MG malignant GBM cells (U87MG) and studied the underlying mechanism of cytotoxic action.

## 2. Results

### 2.1. 5-Aminolevulinic Acid (5-ALA) Inhibits Cell Viability of U87MG Cells

**Figure 1:**
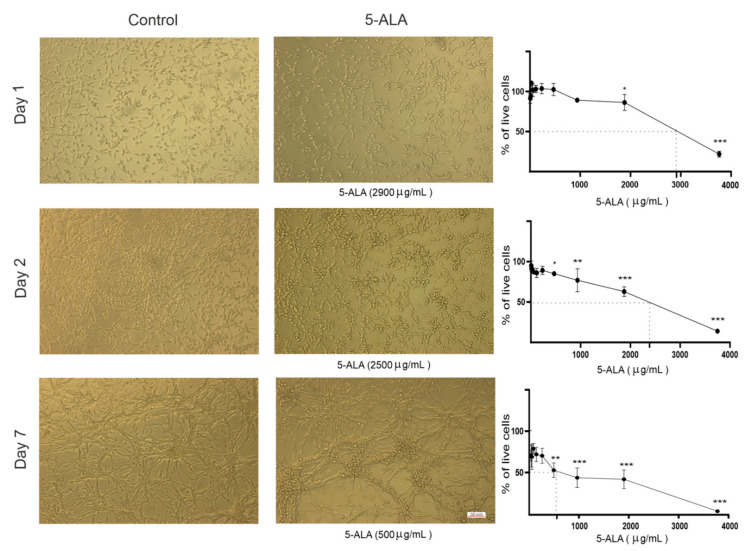
Cytotoxic effects of 5-aminolevulinic acid (5-ALA) on the human U-87 MG malignant GBM cell line (U87MG). Representative phase-contrast micrographs of U87MG cells following 1, 2, and 7 days of treatment with 5-ALA (0–3750 µg/mL) are presented. Cytotoxicity of 5-ALA on the viability of U87MG cells was evaluated using the MTT method. To ascertain the half-maximal inhibitory concentration of 5-ALA, the percentage of live U87MG cells was assessed following 1, 2, and 7 days of treatment with various doses of 5-ALA. The results are presented as means ± standard deviation. *, **, and *** indicate *p* < 0.05, *p* < 0.01, and *p* < 0.001, respectively.

Using MTT assay, the effects of different doses of 5-ALA on cell viability were investigated. To investigate the inhibitory effects of 5-ALA, U87MG cells were treated with different concentrations of 5-ALA at different time intervals (1, 2, and 7 days). As shown in Figure 1, 5-ALA exerted a significant inhibitory effect on the viability of U87MG cells in a time- as well as dose-dependent manner. The half-maximal inhibitory concentration (IC50) of 5-ALA was 2500 μg/mL after 2 days, which was reduced to 500 μg/mL after 7 days (Figure 1).

**Figure 2:**
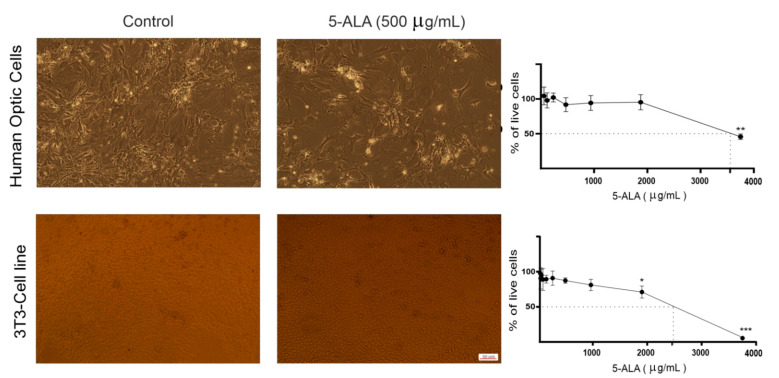
The effect of 5-aminolevulinic acid (5-ALA) on the viability of the human primary optic cells and NIH/3T3 cells. Representative phase-contrast micrographs of primary optic cells obtained from the human optic nerves and NIH/3T3 cells following 7 days of treatment with 5-ALA (500 µg/mL) are shown. Cytotoxicity of 5-ALA (0–3750 µg/mL) on the viability of these non-tumoral cells was evaluated using the MTT assay. To evaluate the half-maximal inhibitory concentration of 5-ALA, the percentage of live cells was assessed following 7 days of treatment with various doses of 5-ALA. The results are presented as means ± standard deviation. *, **, *** Indicate *p* < 0.05, *p* < 0.01, and *p* < 0.001, respectively.

To establish whether 5-ALA was toxic to normal cells, the human optic cells and NIH-3T3 cells were also treated with 5-ALA for 7 days (Figure 2); 5-ALA, at concentrations that were toxic for U87MG cells, was not harmful to the human optic and NIH-3T3 cells.

### 2.2. 5-ALA Induces Apoptosis in U87MG Cells

**Figure 3:**
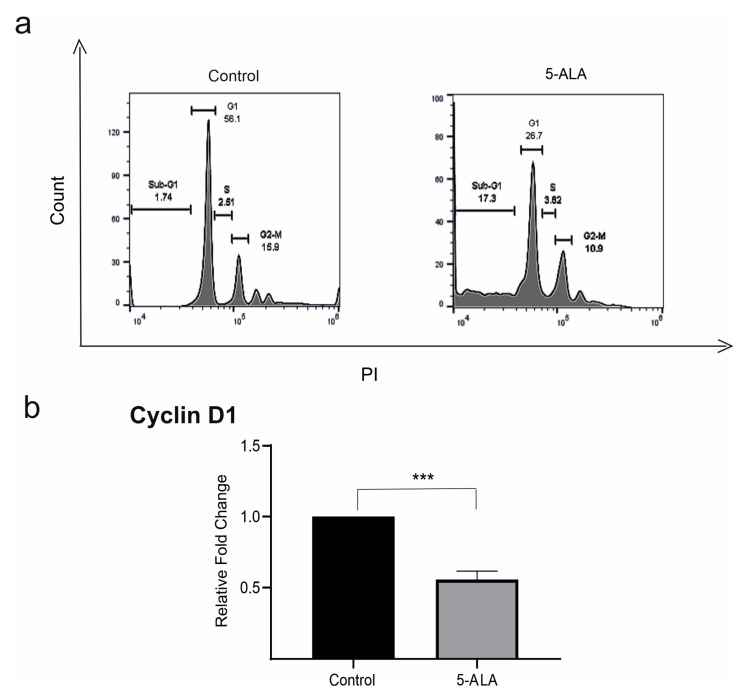
The effects of 5-aminolevulinic acid (5-ALA, 250 µg/mL) on the expression of cyclin D1 and cell cycle of the human U-87 MG malignant GBM cells (U87MG). (**a**) The cell cycle was assessed in U87MG cells incubated with 5-ALA for 7 days and the control group. Note the increased accumulation of U87MG cells in the SUB-G1 population as compared with the control group; (**b**) the application of 5-ALA significantly reduced the expression of cyclin D in U87MG cells as compared with the control group. The results are presented as means ± standard deviation. *** Indicates *p* < 0.001.

To evaluate the mechanisms that 5-ALA inhibited the viability of U87MG cells, both apoptosis and cell cycle in 5-ALA treated cells were evaluated. The assessment of the cell cycle has shown that the application of 5-ALA (250 µg/mL) for 7 days increased the accumulation of U87MG cells in the SUB-G1 population up to 17.3% (Figure 3). Cyclin D1, a modulator of cellular differentiation, plays a critical role in the integration of extracellular signals of various cells in early-to-mid G1 transition [20]. To determine the cell cycle induction, the mRNA expression of cyclin D1 was evaluated. The mRNA expression values of cyclin D1 significantly decreased in 5-ALA treated cells as compared with the control group (Figure 3, *p* < 0.001).

**Figure 4:**
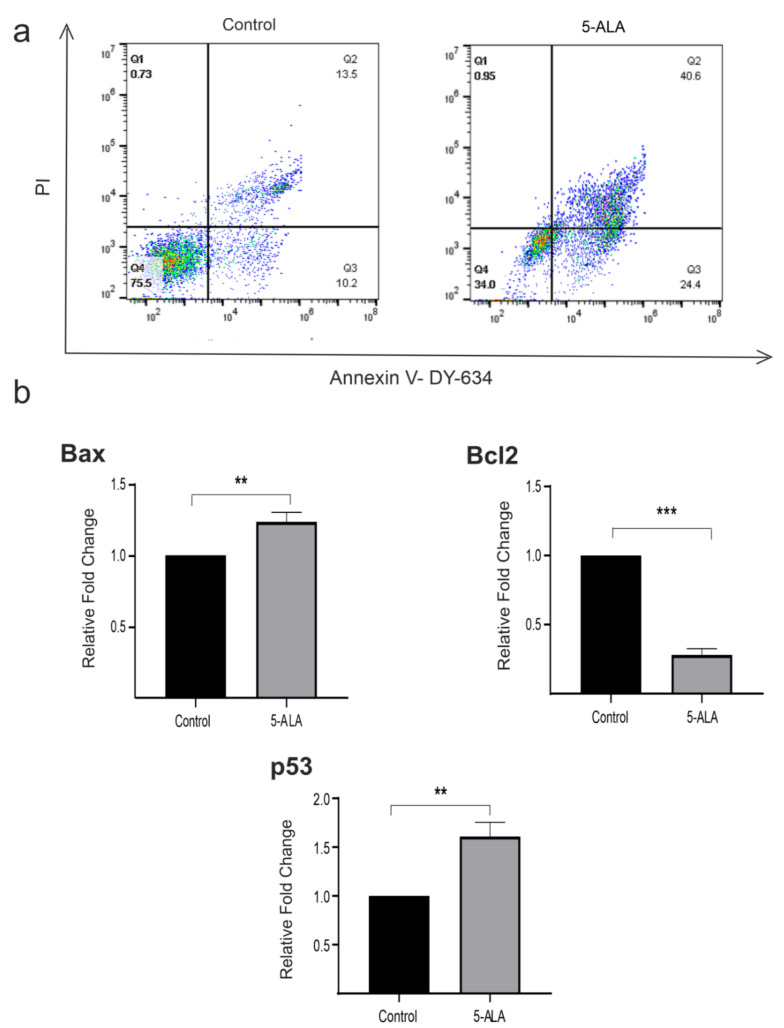
The effects of 5-aminolevulinic acid (5-ALA) on apoptosis and the mRNA expression of various apoptotic biomarkers (p53, Bax, and Bcl-2) of the human U-87 MG malignant GBM cells (U87MG). (**a**) U87MG cells were stained with Annexin V/propidium iodide and evaluated using the flow cytometry technique. U87MG cells were treated with 5-ALA at 250 µg/mL for 7 days. Untreated cells were evaluated as the control group. Diagrams quarter 4 (Q4) to Q1 represent live cells, early apoptotic, late apoptotic, and necrotic cells, respectively. U87MG cells incubated with 5-ALA showed a higher amount of early and late apoptotic cells; (**b**) 5-ALA significantly enhanced the values of Bax and p53, as well as decreased Bcl-2 expression as compared with the control group. The results are presented as means ± standard deviation. **, *** Indicate *p* < 0.01 and *p* < 0.001, respectively.

To detect apoptotic cells, we performed Annexin V-FITC/PI double staining. The analysis of Annexin V/PI double-stained cells has shown that about 40.6% of U87MG cells treated with 5-ALA were in the late apoptotic stage. In contrast, only 13.5% of U87MG cells were in the late apoptotic stage in the control group. Furthermore, we investigated the mRNA expression levels of Bax, Bcl-2, and p53 of cells treated with 5-ALA for 7 days. Treatment of U87MG cells with 5-ALA significantly decreased the expression of Bcl-2 and enhanced the levels of Bax and p53 as compared with the control group (Figure 4, *p* < 0.01).

### 2.3. 5-ALA Increases the Production of ROS

**Figure 5:**
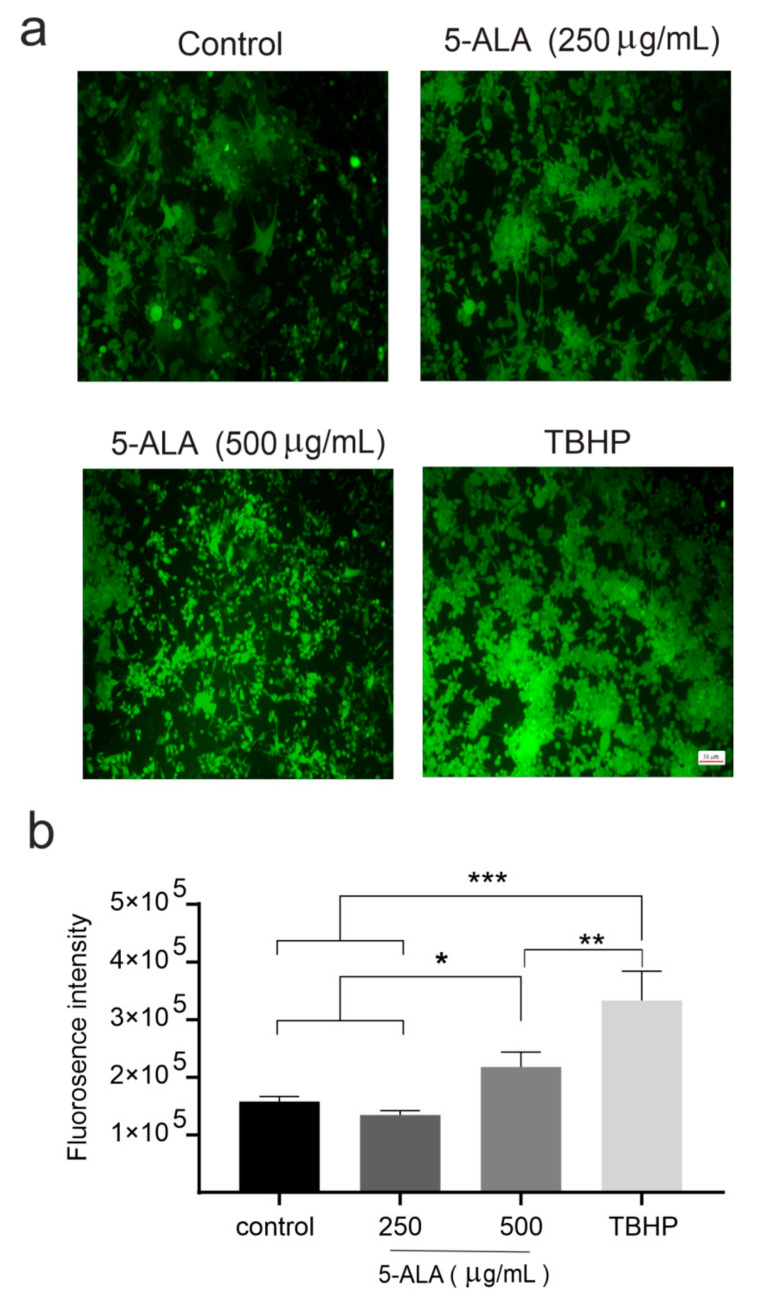
The effects of 5-aminolevulinic acid (5-ALA) on the induction of reactive oxygen species (ROS) in the human U-87 MG malignant GBM cells (U87MG) after 7 days. (**a**) Photomicrographs of ROS generation of U87MG cells in the control, 5-ALA (250 and 500 μg/mL), and tert-butyl hydroperoxide (TBHP) groups were obtained using fluorescence microscopy; (**b**) ROS generation was evaluated by the measurement of fluorescent intensities. Note a significantly greater ROS production after the application of 5-ALA at 500 μg/mL as compared with the cells treated with 5-ALA at 250 μg/mL and the control group. The results are presented as means ± standard deviation. *, **, *** Indicate *p* < 0.05, *p* < 0.01, and *p* < 0.001, respectively.

Previous studies have indicated that 5-ALA-induced PP-IX accumulation enhanced the generation of ROS in different cancerous cells [13]. Our data revealed that 5-ALA in a dose-dependent manner enhanced the generation of ROS in U87MG cells. The values of ROS generation were significantly higher in cells incubated with 500 µg/mL of 5-ALA for 7 days as compared with the cells treated with 5-ALA at 250 µg/mL and the control groups (Figure 5, *p* < 0.05).

### 2.4. 5-ALA Inhibits the Migration of U87MG

**Figure 6:**
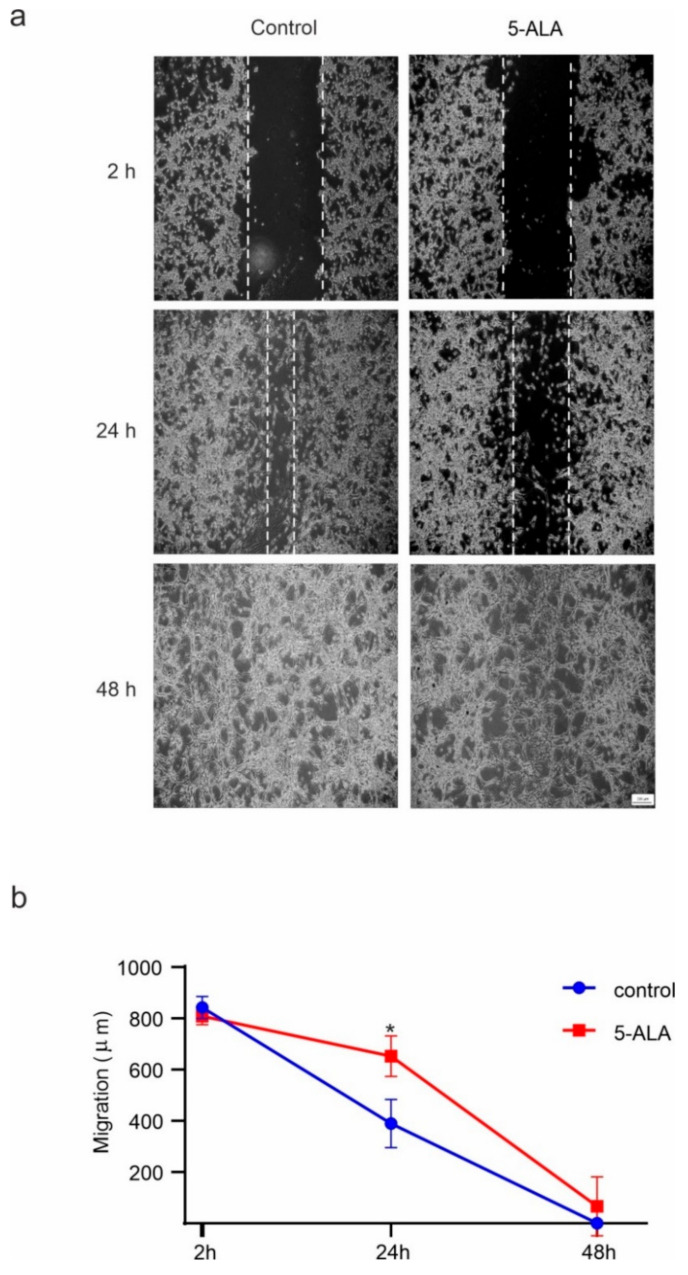
The effects of 5-aminolevulinic acid (5-ALA) on the migration of the human U-87 MG malignant GBM cells (U87MG). The migration of U87MG cells was evaluated by the wound healing approach. Using a micropipette tip, a scratch wound was created through the surface of a confluent monolayer of U87MG cells, and then 5-ALA at 250 µg/mL was applied. The microscopic image were captured after 2, 24, and 48 h of treatment. (**a**) The migration rate of U87MG cells was assessed by the number of cells within the wound area; (**b**) 5-ALA exerted a significant inhibitory effect on the migration of U87MG cells after 24 h as compared with the control group. The results are presented as means ± standard deviation. * Indicates *p* < 0.05.

The wound healing migration model is a standard in vitro method for the evaluation of cell migration and invasiveness [21]. To detect the effect of 5-ALA on the migration of U87MG cells, the wound-healing assay was conducted. As shown in Figure 6, 5-ALA significantly slowed down the migration of U87MG cells after 24 h as compared with the control group (*p* < 0.05). These results suggest that 5-ALA has the potential to inhibit the migration/invasiveness ability of U87MG cells.

## 3. Discussion

The results from the present in vitro study indicated that the long-term application of 5-ALA exhibited an antiproliferative effect on U87MG cells in a time- and concentration-dependent manner. Although administration of 5-ALA after a relatively short period of time and at lower doses did not exert cytotoxic effects on U87MG cells, it exerted moderate antitumoral effects over a longer period of time and at higher concentrations; 5-ALA reduced the viability of U87MG cells through the induction of cell arrest and apoptosis. Our data indicate that the upregulation of Bax, p53, and ROS, as well as the downregulation of Bcl-2, are involved in the antitumoral effect of 5-ALA on U87MG cells. Furthermore, 5-ALA at concentrations that inhibited the growth of U87MG cells is likely a safe substance with little impact on normal cells.

Treatment of U87MG cells with chemotherapeutic drugs, such as temozolomide, or ionizing radiation has been shown to lead to a significant reduction of cell viability in a dose-dependent manner [22,23], possibly via increasing the expression of P53, Bax, and ROS generation as well as decreasing the expression of Bcl-2 [24,25]. Dysregulation of the p53 tumor suppressor pathway has been observed in approximately 90% of GBM patients, which was associated with a poor prognosis [26]. Bcl-2 overexpression has also been reported in about 50% of GBM patients, which was associated with a significant enhancement of tumor resistance against adjuvant therapies [27]. Among pro-apoptotic inducers, the activation of Bax protein inhibits the proliferation and self-renewal of GBM cells [28]. Apoptosis is a key mechanism for the maintenance of cell proliferation and cell death in GBM [29], and targeting apoptosis represents an optimal therapeutic strategy for GBM [30]. Studies have shown that 5-ALA increases apoptotic cell death in resistant prostate cancer cells [31] and enhancement of intracellular PP-IX induces p53-dependent apoptosis in lymphocytic leukemia cells [32]. A reduction in cell viability following the application of 5-ALA and photodynamic therapy has been attributed to cell apoptosis via the increasing p53 protein level as well as Bax/Bcl-2 ratio [33,34]. The cotreatment of 5-ALA and photodynamic therapy leads to an increase in the Bax/Bcl-2 ratio and stimulates the caspase cascade by the activation of caspase-3 and caspase-9 [10]. In contrast to our findings, some investigations did not report the cytotoxic effects of 5-ALA on U87MG cells [35,36]. This discrepancy could be due to the application of lower doses or/and a shorter incubation period of 5-ALA with cancer cells. Cell cycle arrest can result in either cell death, usually through the activation of apoptosis, or an effective DNA repair, which may prevent the transformation of normal cells into neoplastic cells [37]. The interaction of cell cycle regulators and apoptotic mediators, such as P53, plays an important role in the modulation of tumor homeostasis [38]. In our study, the application of 5-ALA on U87MG cells led to a significantly greater accumulation of tumor cells in the sub-G1 phase. The P53-mediated cell cycle arrest and apoptosis in tumor cells are predicted to be favorable outcomes of tumor therapy [39]. Furthermore, our data indicated that 5-ALA could induce apoptosis via the regulation of proapoptotic or pro-survival of Bcl-2 family members, modulation of p53-mediated transcriptional activity, and alteration of ROS activity; 5-ALA inhibited cell migration and modulated the production of the apoptotic pathways at lower concentrations than IC50. Lower doses of 5-ALA were tested in these experiments to reduce the drug cytotoxicity for a better evaluation of cell behaviors.

Furthermore, 5-ALA in combination with photodynamic therapy has been shown to induce apoptosis and arrest the cell cycle at the G0/G1 phase in esophageal cancer [40]. Accumulation of PP-IX in neoplastic cells has been shown to induce mitochondrial membrane depolarization, activate mitochondria-to-nuclear translocation of the transcription factors, and consequently lead to DNA damage [41]. Targeting the cell-cycle machinery, particularly mitotic proteins, represents a promising therapeutic strategy to combat GBM [42]. The expression of cyclin D1 has been shown to be directly correlated with the progression and dissemination of GBM [43,44]. Inhibition of cyclin D1 has resulted in enhanced sensitivity of glioma cells to temozolomide, pointing to its key role in the chemoresistance of GBM cells [45]. In our study, 5-ALA reduced the expression of cyclin D1 and inhibited the migration of GBM cells.

As compared with chemotherapeutic agents, 5-ALA leads to the accumulation of PP-IX preferentially in cancerous cells, and thus damage to the surrounding healthy brain tissues is limited [46]. Our in vitro study indicates that 5-ALA at cytotoxic concentrations for U87MG cells is safe for normal cells, including the optic nerve (consists of ganglion cell axons, astroglial cells, oligodendrocytes, and glial cells) and NIH/3T3 cells. However, further in vitro and in vivo studies are required to demonstrate the safety of long-term application of 5-ALA for nontumoral cells. Contrary to our data, it has been shown that 5-ALA promoted the production of ROS and induction of apoptosis through activation of p53 and caspases in gastric normal cells but not in gastric cancer cells [47].

The present report has some limitations. We examined antitumor effects of 5-ALA on the cell line U87MG, which may not be representative of various types of highly malignant glioma. Nevertheless, the present study can be of great help for future in vitro and in vivo investigations deciphering the antitumoral effects of 5-ALA in GBM. Furthermore, this study is limited by the lack of information on the correlation of antitumor effects of 5-ALA with the augmentation of intracellular PP-IX in tumor cells. Further experimentation on the effect of 5-ALA alone or in combination with other substances or therapies that enhances intracellular PP-IX on tumor cells is required. It has been shown that several compounds, such as vitamin D, ciprofloxacin, deferiprone, and febuxostat, as well as different adjuvant therapies, such as photodynamic therapy and ionizing irradiation, augment the cytotoxicity of 5-ALA in tumoral cells [48].

Taken together, the present study is the first to indicate that 5-ALA can target the viability and migration of U87MG cells, presumably via the induction of cell cycle arrest and apoptosis, enhancement of ROS generation, and inhibition of cyclin D1. However, our conclusion relies only on in vitro studies, and additional in vivo experiments are needed to demonstrate the antitumoral efficacy of 5-ALA on GBM cells and to validate its possible clinical application.

## 4. Materials and Methods

To evaluate the antitumor effect of 5-ALA, U87MG cells were cultured and cultivated with different doses of 5-ALA. The effects of 5-ALA on different properties of U87MG cells were evaluated. This study was approved by the Ethical Committee of Shefa Neuroscience Research Centre, Tehran, Iran. Informed consent has been obtained from the patients.

### 4.1. Cell Cultures

U87MG and NIH/3T3 cells were obtained from the National Cell Bank of Iran, Pasteur Institute, Tehran, Iran. To establish particular requirements for cell lines, we prepared the flasks, labeled with cell line name, passage number, and date. Using appropriate personal protective equipment, we collected an ampoule of cells from liquid nitrogen and transferred them to the laboratory on dry ice. Then, frozen cells were thawed rapidly (1–2 min) in a water bath (37 °C). The thawed cells were slowly diluted with a warmed growth medium before incubation. The cells were plated at high density in high-glucose Dulbecco’s modified Eagle’s medium (DMEM; Gibco, Karlsruhe, Germany). Then, streptomycin-penicillin and 10% fetal bovine serum (FBS, 100 μg/mL) were added (Gibco, Karlsruhe, Germany) to the medium. As controls, optic nerves were obtained from two patients with traumatic optic neuropathy. The primary optic cells were grown in DMEM/F12 medium containing 10% FBS, 1% L-glutamate (Gibco, Karlsruhe, Germany), 20 ng/mL epidermal growth factor (Sigma, Darmstadt, Germany), 100 μg/mL streptomycin (Gibco, Karlsruhe, Germany), and 100 U/mL penicillin (Gibco, Karlsruhe, Germany). A humidified incubator (5% CO_2_) was used to culture cells at 37 °C. The optic nerve consists of various cells, including ganglion cell axons, astroglial cells, oligodendrocytes, glial cells, blood vessels, and connective tissue [49]. Dissociated cell cultures from the optic nerve contain astrocytes and oligodendrocytes [50,51].

### 4.2. Evaluation of Cell Viability

As described previously [52], a colorimetric MTT assay (Atocel, Graz, Austria) was used to determine cellular metabolic activity. Briefly, U87MG, NIH/3T3, and primary optic cells were plated in 96-well plates (5000 cells per well) and cultured overnight. The cells were incubated with various concentrations of 5-ALA (0–3750 µg/mL, Gliolan, Wedel, Germany) for 1, 2, and 7 days. Every three days, the medium was changed, and 5-ALA was added with the same corresponding concentrations. Then, each well was washed twice with PBS to remove the 5-ALA and the MTT solution was added to the wells. Afterward, dimethyl sulfoxide (DMSO, 100 µL, Sigma, Darmstadt, Germany) was added to each well and incubated for 3 h to disintegrate the formazan crystals in DMSO. The absorbance at 545 and 630 nm was measured on a Stat FAX303 plate reader (Awareness Technologies, Westport, CT, USA). Cells were largely protected from direct exposure to light sources by keeping cell culture dishes in a light-protected humidified chamber, using aluminum foil during dish transport, and performing the measurements in dim light.

### 4.3. Evaluation of Apoptosis

The apoptosis rate of U87MG cells treated with 5-ALA was examined using Annexin V-DY-634 PI kit (Abcam, Cambridge, UK). Briefly, 10^5^ cells were seeded in 6-well plates and treated for 7 days with 5-ALA (250 µg/mL). The culture medium was replaced every 3 days. Next, cells were trypsinized and washed two times with PBS. Then, 100 µL of Annexin V-DY-634 PI was added to each sample. After 10 min of incubation in the dark at room temperature, 400 µL of 1× buffer was added to the samples. Flow cytometry analysis was performed using a BD Facscalibur™ flow cytometer (Becton Dickinson, Heidelberg, Germany).

### 4.4. RNA Analysis and Quantitative Reverse Transcription (qRT)-PCR

Total RNA was extracted from the treated cells with 5-ALA (250 µg/mL) for 7 days by the RNeasy Mini Kit (Qiagen, Hilden, Germany). Then, the RNA was reverse-transcribed using RevertAid First Strand cDNA Synthesis (Thermo Fisher Scientific, Wesel, Germany) and qRT-PCR with specific primers for glyceraldehyde 3-phosphate dehydrogenase (GAPDH), Bax, Bcl-2, p53, and cyclin D1 (Table 1) was accomplished using RealQ Plus 2X MasterMix Green-without Rox™ (Ampliqon, Odense, Denmark). A LightCycler^®^ 96 Instrument system (Roche life Science, Indianapolis, IN, USA) was used for gene amplification, under the following conditions: 95 °C for 15 min, 40 cycles of 95 °C for 30 s, and annealing/extension at 60 °C for 60 s. The melting curve was generated at 60–95 °C for 6 s. GADPH was applied to normalize alterations in gene expressions. Using the 2−ΔΔCq technique, the fold change in gene expression was assessed [53].

### 4.5. Assessment of ROS Generation

The cellular ROS detection kit (Abcam, Cambridge, UK) was used to evaluate the generation of ROS. The 25 × 10^3^ U87MG cells were seeded and cultivated overnight. After 24 h, the U87MG cells were washed with PBS and incubated with dichloro-dihydro-fluorescein diacetate (25 μM, H2DCFDA; Santa Cruz, Heidelberg, Germany) for 45 min in the dark. Afterward, the cells were rewashed and treated with 5-ALA at 250 and 500 µg/mL. The fluorescence was measured (excitation/emission 485/535 nm) using a Victor X5 Multiplable Plate Reader (Perkin Elmer, Waltham, MA, USA). Tert-butyl hydroperoxide (150 µM), a selective inhibitor of mitochondrial respiratory chain enzyme, was used as a positive control. Dissociated U87MG cells were inserted on poly-L-lysine coated 96-well plates. Then, the cells were washed with PBS, and H2DCFDA fluorescence was added to the medium and incubated for 30 min. The 5-ALA-induced ROS activity was evaluated by fluorescent microscopy. Images were obtained using a fluorescent microscope (Axiovert 200, Zeiss, Berlin, Germany).

### 4.6. Evaluation of Cell Cycle

To evaluate the cell cycle phase of U87MG cells, 10^5^ cells were treated with 5-ALA (250 µg/mL) for 7 days at 37 °C. The medium was changed every 3 days. Then, DNA content analysis was performed with PI staining (Abcam, Cambridge, UK). Briefly, cells were trypsinized and centrifuged at 3000 rpm for 5 min at 4 °C. Next, the cells were suspended in ice-cold PBS and fixed with ethanol (70%) for 2 h, at 4 °C. After fixation, U87MG cells were washed and resuspended with ice-cold PBS. Afterward, U87MG cells were incubated with RNase A (100 μL) for 30 min at room temperature, and then were resuspended in 400 μL PI-Triton X-100 solution (contained 1 mg/mL sodium citrate, 50 μg/mL PI, and 0.1% Triton X-100) for another 30 min in the dark. Afterward, cell cycle distribution was investigated in a BD FACSCALIBUR™ FLOW CYTOMETER (Becton Dickinson, Heidelberg, Germany). Data analysis was conducted by the FlowJo V10 software (FlowJo, Ashland, OR, USA).

### 4.7. Evaluation of Migration Property

U87MG cell migration was examined by the wound-healing method [54,55,56]. After 48 h of incubation, the U87MG cells reached 90% confluence. A straight scratch was created on the cell monolayer using the tip of a sterile pipette (100 μL). The cell debris was removed by PBS washing. Then, the cells were treated with 5-ALA (250 μg/mL) and incubated for 48 h. The border of the scratch was detected by an inverted microscope (Axiovert 200, Zeiss, Germany). U87MG cell migration distance was assessed after 2, 24, and 48 h.

### 4.8. Statistical Analysis

Data are expressed as mean ± standard error. The statistical analysis was conducted by a one-way analysis of variance (ANOVA) followed by Dunnett’s post hoc test, and a two-tailed Student’s paired t-test. The *p*-value of less than 0.05 was considered significant.

## Figures and Tables

**Table 1:** Primers used in this study.

Gene Symbol	Gene Name	Primers (5′ → 3′)	Accession Number
*Bax*	Bcl-2-associated X protein	Forward: TGACGGCAACTTCAACTGGG Reverse: CTTCAGTGACTCGGCCAGGG	NM_001291428.2
*Bcl-2*	B-cell lymphoma 2	Forward: GTCATGTGTGTGGAGAGCGTC Reverse: CCGTACAGTTCCACAAAGGCATC	NM_000633.3
*P53*	Tumor suppressor protein	Forward: ACACGCTTCCCTGGATTGG Reverse: CTAGGATCTGACTGCGGCTC	NM_000546.6
*CCND1*	Cyclin D1	Forward: CAAATGTGTGCAGAAGGAGGTC Reverse: CTCGCACTTCTGTTCCTCGC	NM_053056.3
*GAPDH*	Glyceraldehyde-3-phosphate dehydrogenase	Forward: TCAAGATCATCAGCAATGCCTCC Reverse: GCCATCACGCCACAGTTTC	NM_001357943.2

## Data Availability

All data will be made available upon reasonable request.

## Abbreviations

5-ALA	5-Aminolevulinic acid
DMEM	Dulbecco’s modified Eagle’s medium
DMSO	Dimethyl sulfoxide
FBS	Fetal bovine serum
GBM	Glioblastoma multiforme
IC50	Half-maximal inhibitory concentration
PP-IX	Protoporphyrin IX
ROS	Reactive oxygen species
U87MG	U-87 malignant GBM cell line

## References

[1] Koshy M., Villano J.L., Dolecek T.A., Howard A., Mahmood U., Chmura S.J., Weichselbaum R.R., McCarthy B.J. (2012). Improved survival time trends for glioblastoma using the SEER 17 population-based registries. J. Neuro Oncol..

[2] Witthayanuwat S., Pesee M., Supaadirek C., Supakalin N., Thamronganantasakul K., Krusun S. (2018). Survival Analysis of Glioblastoma Multiforme. Asian Pac. J. Cancer Prev..

[3] Han S.J., Englot D.J., Birk H., Molinaro A.M., Chang S.M., Clarke J.L., Prados M.D., Taylor J.W., Berger M.S., Butowski N.A. (2015). Impact of Timing of Concurrent Chemoradiation for Newly Diagnosed Glioblastoma. Neurosurgery.

[4] Stummer W., Pichlmeier U., Meinel T., Wiestler O.D., Zanella F., Reulen H.-J. (2006). Fluorescence-guided surgery with 5-aminolevulinic acid for resection of malignant glioma: A randomised controlled multicentre phase III trial. Lancet Oncol..

[5] Kuhnt D., Becker A., Ganslandt O., Bauer M., Buchfelder M., Nimsky C. (2011). Correlation of the extent of tumor volume resection and patient survival in surgery of glioblastoma multiforme with high-field intraoperative MRI guidance. Neuro-Oncology.

[6] Hadjipanayis C.G., Stummer W. (2019). 5-ALA and FDA approval for glioma surgery. J. Neuro Oncol..

[7] Armocida D., Pesce A., Di Giammarco F., Frati A., Santoro A., Salvati M. (2019). Long Term Survival in Patients Suffering from Glio-blastoma Multiforme: A Single-Center Observational Cohort Study. Diagnostics.

[8] Walter S., Susanne S., Simon W., Herbert S., Clemens F., Claudia G., Alwin E.G., Rainer K., Hans J.R. (1998). Intraoperative Detection of Malignant Gliomas by 5-Aminolevulinic Acid-induced Porphyrin Fluorescence. Neurosurgery.

[9] Nakayama T., Otsuka S., Kobayashi T., Okajima H., Matsumoto K., Hagiya Y., Inoue K., Shuin T., Nakajima M., Tanaka T. (2016). Dormant cancer cells accumulate high protoporphyrin IX levels and are sensitive to 5-aminolevulinic acid-based photodynamic therapy. Sci. Rep..

[10] Karmakar S., Banik N.L., Patel S.J., Ray S.K. (2007). 5-Aminolevulinic acid-based photodynamic therapy suppressed survival factors and activated proteases for apoptosis in human glioblastoma U87MG cells. Neurosci. Lett..

[11] Coupienne I.C.I., Bontems S., Dewaele M., Rubio N., Habraken Y., Fulda S., Agostinis P., Piette J. (2011). NF-kappaB inhibition improves the sensitivity of human glioblastoma cells to 5-aminolevulinic acid-based photodynamic therapy. Biochem. Pharmacol..

[12] Yamamoto J., Ogura S.-I., Shimajiri S., Nakano Y., Akiba D., Kitagawa T., Ueta K., Tanaka T., Nishizawa S. (2014). 5-Aminolevulinic acid-induced protoporphyrin IX with multi-dose ionizing irradiation enhances host antitumor response and strongly inhibits tumor growth in experimental glioma in vivo. Mol. Med. Rep..

[13] Yamada K., Murayama Y., Kamada Y., Arita T., Kosuga T., Konishi H., Morimura R., Shiozaki A., Kuriu Y., Ikoma H. (2019). Radiosensitizing effect of 5-aminolevulinic acid in colorectal cancer in vitro and in vivo. Oncol. Lett..

[14] Yamamoto J., Ogura S.-I., Tanaka T., Kitagawa T., Nakano Y., Saito T., Takahashi M., Akiba D., Nishizawa S. (2012). Radiosensitizing effect of 5-aminolevulinic acid-induced protoporphyrin IX in glioma cells in vitro. Oncol. Rep..

[15] Miyake M., Tanaka N., Hori S., Ohnishi S., Takahashi H., Fujii T., Owari T., Ohnishi K., Iida K., Morizawa Y. (2019). Dual benefit of supplementary oral 5-aminolevulinic acid to pelvic radiotherapy in a syngenic prostate cancer model. Prostate.

[16] Yang X., Palasuberniam P., Myers K.A., Wang C., Chen B. (2016). Her2 oncogene transformation enhances 5-aminolevulinic acid-mediated protoporphyrin IX production and photodynamic therapy response. Oncotarget.

[17] Yoshioka E., Chelakkot V.S., Licursi M., Rutihinda S.G., Som J., Derwish L., King J.J., Pongnopparat T., Mearow K., Larijani M. (2018). Enhancement of Cancer-Specific Protoporphyrin IX Fluorescence by Targeting Oncogenic Ras/MEK Pathway. Theranostics.

[18] Kitajima Y., Ishii T., Kohda T., Ishizuka M., Yamazaki K., Nishimura Y., Tanaka T., Dan S., Nakajima M. (2019). Mechanistic study of PpIX accumulation using the JFCR39 cell panel revealed a role for dynamin 2-mediated exocytosis. Sci. Rep..

[19] Luwor R., Morokoff A.P., Amiridis S., D’Abaco G., Paradiso L., Stylli S.S., Nguyen H.P.T., Tarleton M., Young K.A., O’Brien T.J. (2019). Targeting Glioma Stem Cells by Functional Inhibition of Dynamin 2: A Novel Treatment Strategy for Glioblastoma. Cancer Investig..

[20] Koeller H.B., Ross M.E., Glickstein S.B. (2008). Cyclin D1 in excitatory neurons of the adult brain enhances kainate-induced neurotoxicity. Neurobiol. Dis..

[21] Friedl P., Wolf K. (2009). Plasticity of cell migration: A multiscale tuning model. J. Cell Biol..

[22] Fisher T., Galanti G., Lavie G., Jacob-Hirsch J., Kventsel I., Zeligson S., Winkler R., Simon A.J., Amariglio N., Rechavi G. (2007). Mechanisms Operative in the Antitumor Activity of Temozolomide in Glioblastoma Multiforme. Cancer J..

[23] Paolini A., Pasi F., Facoetti A., Mazzini G., Corbella F., Di Liberto R., Nano R. (2011). Cell death forms and HSP70 expression in U87 cells after ionizing radiation and/or chemotherapy. Anticancer. Res..

[24] Hirose Y., Berger M.S., Pieper R.O. (2001). Abrogation of the Chk1-mediated G(2) checkpoint pathway potentiates te-mozolomide-induced toxicity in a p53-independent manner in human glioblastoma cells. Cancer Res..

[25] Akbarnejad Z., Eskandary H., Dini L., Vergallo C., Nematollahi-Mahani S.N., Farsinejad A., Abadi M.F.S., Ahmadi M. (2017). Cytotoxicity of temozolomide on human glioblastoma cells is enhanced by the concomitant exposure to an extremely low-frequency electromagnetic field (100 Hz, 100 G). Biomed. Pharmacother..

[26] Zhang Y., Dube C., Gibert J.M., Cruickshanks N., Wang B., Coughlan M., Yang Y., Setiady I., Deveau C., Saoud K. (2018). The p53 Pathway in Glioblastoma. Cancers.

[27] Fels C., Schäfer C., Hüppe B., Bahn H., Heidecke V., Kramm C.M., Lautenschläger C., Rainov N.G. (2000). Bcl-2 expression in higher-grade human glioma: A clinical and experimental study. J. Neuro Oncol..

[28] Daniele S., Pietrobono D., Costa B., Giustiniano M., La Pietra V., Giacomelli C., La Regina G., Silvestri R., Taliani S., Trincavelli M.L. (2017). Bax Activation Blocks Self-Renewal and Induces Apoptosis of Human Glioblastoma Stem Cells. ACS Chem. Neurosci..

[29] Valdés-Rives S.A., Casique-Aguirre D., Germán-Castelán L., Velasco-Velázquez M.A., González-Arenas A. (2017). Apoptotic Signaling Pathways in Glioblastoma and Therapeutic Implications. BioMed Res. Int..

[30] Eisele G., Weller M. (2013). Targeting apoptosis pathways in glioblastoma. Cancer Lett..

[31] Teper E., Makhov P., Golovine K., Canter D.J., Myers C.B., Kutikov A., Sterious S.N., Uzzo R.G., Kolenko V.M. (2012). The Effect of 5-Aminolevulinic Acid and Its Derivatives on Protoporphyrin IX Accumulation and Apoptotic Cell Death in Castrate-resistant Prostate Cancer Cells. Urology.

[32] Jiang L., Malik N., Acedo P., Zawacka-Pankau J. (2019). Protoporphyrin IX is a dual inhibitor of p53/MDM2 and p53/MDM4 interactions and induces apoptosis in B-cell chronic lymphocytic leukemia cells. Cell Death Discov..

[33] Chang M., Ma X., Ouyang T., Lin J., Liu J., Xiao Y., Chen H., Yu J., Huang Y., Xu M. (2015). Potential Molecular Mechanisms Involved in 5-Aminolevulinic Acid–Based Photodynamic Therapy against Human Hypertrophic Scars. Plast. Reconstr. Surg..

[34] Wei X.Q., Ma H.Q., Liu A.H., Zhang Y.Z. (2013). Synergistic Anticancer Activity of 5-Aminolevulinic Acid Photodynamic Therapy in Combination with Low-dose Cisplatin on Hela Cells. Asian Pac. J. Cancer Prev..

[35] Suehiro S., Ohnishi T., Yamashita D., Kohno S., Inoue A., Nishikawa M., Ohue S., Tanaka J., Kunieda T. (2018). Enhancement of antitumor activity by using 5-ALA–mediated sonodynamic therapy to induce apoptosis in malignant gliomas: Significance of high-intensity focused ultrasound on 5-ALA-SDT in a mouse glioma model. J. Neurosurg..

[36] Sheehan K., Sheehan D., Sulaiman M., Padilla F., Moore D., Sheehan J., Xu Z. (2020). Investigation of the tumoricidal effects of sonodynamic therapy in malignant glioblastoma brain tumors. J. Neuro Oncol..

[37] Wiman K.G., Zhivotovsky B. (2017). Understanding cell cycle and cell death regulation provides novel weapons against human diseases. J. Intern. Med..

[38] Pucci B., Kasten M., Giordano A. (2000). Cell Cycle and Apoptosis. Neoplasia.

[39] Chen J. (2016). The Cell-Cycle Arrest and Apoptotic Functions of p53 in Tumor Initiation and Progression. Cold Spring Harb. Perspect. Med..

[40] Chen X., Zhao P., Chen F., Li L., Luo R. (2010). Effect and mechanism of 5-aminolevulinic acid-mediated photodynamic therapy in esophageal cancer. Lasers Med. Sci..

[41] Li Q., Wang X., Zhang K., Li X., Liu Q., Wang P. (2013). DNA Damage and Cell Cycle Arrest Induced by Protoporphyrin IX in Sarcoma 180 Cells. Cell. Physiol. Biochem..

[42] Castro-Gamero A.M., Pezuk J.A., Brassesco M.S., Tone L.G. (2018). G2/M inhibitors as pharmacotherapeutic opportunities for glioblastoma: The old, the new, and the future. Cancer Biol. Med..

[43] Cemeli T., Guasch-Vallés M., Nàger M., Felip I., Cambray S., Santacana M., Gatius S., Pedraza N., Dolcet X., Ferrezuelo F. (2019). Cytoplasmic cyclin D1 regulates glioblastoma dissemination. J. Pathol..

[44] Mahzouni P., Taheri F. (2019). An Immunohistochemical Study of Cyclin D1 Expression in Astrocytic Tumors and its Correlation with Tumor Grade. Iran. J. Pathol..

[45] Zhang D., Dai D., Zhou M., Li Z., Wang C., Lu Y., Li Y., Wang J. (2018). Inhibition of Cyclin D1 Expression in Human Glioblastoma Cells is Associated with Increased Temozolomide Chemosensitivity. Cell. Physiol. Biochem..

[46] Wang W., Tabu K., Hagiya Y., Sugiyama Y., Kokubu Y., Murota Y., Ogura S.-I., Taga T. (2017). Enhancement of 5-aminolevulinic acid-based fluorescence detection of side population-defined glioma stem cells by iron chelation. Sci. Rep..

[47] Ito H., Kurokawa H., Suzuki H., Indo H.P., Majima H.J., Matsui H. (2019). 5-Aminolevulinic acid induced apoptosis via oxidative stress in normal gastric epithelial cells. J. Clin. Biochem. Nutr..

[48] Kast R.E., Skuli N., Sardi I., Capanni F., Hessling M., Frosina G., Kast A.P., Karpel-Massler G., Halatsch M.E. (2018). Augmentation of 5-Aminolevulinic Acid Treatment of Glioblastoma by Adding Ciprofloxacin, Deferiprone, 5-Fluorouracil and Febuxostat: The CAALA Regimen. Brain Sci..

[49] Triviño A., Ramírez J.M., Salazar J.J., Ramírez A.I., Castellano B., González B., Nieto-Sampedro M. (1998). Astroglial Architecture of the Human Optic Nerve: Functional Role of Astrocytes.

[50] Kennedy P., Lisak R. (1980). Astrocytes and oligodendrocytes in dissociated cell culture of adult rat optic nerve. Neurosci. Lett..

[51] Yang P., Hernandez M. (2003). Purification of astrocytes from adult human optic nerve heads by immunopanning. Brain Res. Protoc..

[52] Jahan-Abad A.J., Karima S., Negah S.S., Noorbakhsh F., Borhani-Haghighi M., Gorji A. (2019). Therapeutic potential of conditioned medium derived from oligodendrocytes cultured in a self-assembling peptide nanoscaffold in experimental autoimmune encephalomyelitis. Brain Res..

[53] Livak K.J., Schmittgen T.D. (2001). Analysis of relative gene expression data using real-time quantitative PCR and the 2^–ΔΔCT^ Method. Methods.

[54] Sahab-Negah S., Ariakia F., Jalili-Nik M., Afshari A.R., Salehi S., Samini F., Rajabzadeh G., Gorji A. (2020). Curcumin Loaded in Niosomal Nanoparticles Improved the Anti-tumor Effects of Free Curcumin on Glioblastoma Stem-like Cells: An In Vitro Study. Mol. Neurobiol..

[55] Grada A., Otero-Vinas M., Prieto-Castrillo F., Obagi Z., Falanga V. (2017). Research Techniques Made Simple: Analysis of Collective Cell Migration Using the Wound Healing Assay. J. Investig. Dermatol..

[56] Rodriguez L.G., Wu X., Guan J.-L. (2005). Wound-Healing Assay. Cell Migration. Methods in Molecular Biology.

